# The Impact of Distance and Altitude on Railway Environmental Noise Based on Cerebral Oxygenated Hemoglobin Saturation

**DOI:** 10.3390/brainsci15050439

**Published:** 2025-04-24

**Authors:** Min-kyeong Kim, Duckshin Park

**Affiliations:** 1 Railroad Test & Certification Division, Korea Railroad Research Institute (KRRI), Cheoldo Bangmulgwanro, Uiwang-si 16105, Republic of Korea; 2 Transportation Environmental Research Department, Korea Railroad Research Institute (KRRI), Cheoldo Bangmulgwanro, Uiwang-si 16105, Republic of Korea; dspark@krri.re.kr

**Keywords:** fNIRS, brain activation, railway environmental noise, stress quantification, GLM

## Abstract

Railways are considered an environmentally sustainable mode of transportation but can pose significant environmental challenges due to their operation and associated activities. Among these, noise generation is a persistent source of public complaints. In Korea, a maximum distance of 100 m from buildings has been proposed for new railway developments in residential areas, although this guideline lacks a solid foundation based on experimental evidence. Noise barriers are often installed as a mitigation measure; however, there is no standardized guideline for their height in relation to their effectiveness at varying distances. The distances and altitudes set in this study took into account accessibility and the height of noise barriers on actual railway sites. In particular, we examined the effects of altitude above and distance from a railway site under the assumption that the prefrontal cortex would be physiologically affected by noise exposure. In this study, we conducted the first analysis in Korea of cerebral blood flow changes in response to noise, to assess quantitatively the stress effects caused by railway environmental noise at varying distances from, and altitudes above, a railway. Using functional near-infrared spectroscopy (fNIRS), we measured prefrontal cortex activation in 10 adult males (average age: 33.2 years). Brain activation was evaluated under different distances from (40 and 100 m) and altitudes above (1st and 4th floors of a building) a railway through a paired-sample *t*-test analysis. Discomfort was felt at relatively close distances to the railway, and there were no differences in perceived discomfort between the examined floors. Brain activation due to environmental noise was highest in channel 43 (left DLPFC) for altitude (floor) and in channel 37 (left FPC) for distance. Significant differences in activation were observed in the corresponding Brodmann areas, varying based on altitude and distance (*p* < 0.05). These results provide valuable scientific data for the preliminary design phase of new railway developments, particularly with regard to determining appropriate residential distance and noise barrier specifications, to enhance comfort of nearby residents. Furthermore, they may contribute to the improvement of quality of life by reducing stress caused by railway environmental noise.

## 1. Introduction

Noise pollution is a significant environmental issue in modern society, with noise exposure steadily increasing due to the ongoing urbanization and industrialization. Noise generated from transportation infrastructure, such as railways, can have a negative impact on the surrounding environment and residents [1,2,3]. Environmental noise is a significant concern associated with railway development, often leading to public complaints. To address this issue, in Korea, the maximum distance for a new railway development from residential properties has been set to 100 m [4,5]. Noise barriers have also been installed in locations where noise from railways is severe; however, there is no standardized guideline for their height in relation to their effectiveness at varying distances from the railway [4,5].

The World Health Organization (WHO) recognizes that noise exposure is associated with various health problems, such as psychological stress, sleep disorders, and cardiovascular diseases [6]. Railway environmental noise can have a serious impact on daily life due to its continuous and periodic nature [1,2,3,6,7]. Noise stress can lead to physiological changes beyond simple discomfort, but most previous studies of railway environmental noise have focused on auditory responses or general psychological effects, or only on noise generated by railway vehicles. There has been little quantitative analysis of the effects of noise stress on specific brain regions, especially the frontal lobe, which plays an important role in cognitive processes and emotional regulation. Functional near-infrared spectroscopy (fNIRS) can noninvasively observe brain activity by measuring blood flow changes and oxygenation status and is therefore a very useful method for understanding physiological responses [7,8,9,10,11,12,13,14,15]. Additionally, environmental variables such as altitude and distance can provide specific criteria for the height and location of noise barrier installations. It is therefore crucial to investigate these factors to understand more fully the specific effects of railway environmental noise on the brain’s frontal lobe function. Noise is an environmental stressor, and stress can be a trigger for mental illness. There are studies that show that exposure to high-intensity noise is associated with psychiatric symptoms such as headaches, nausea, emotional instability, argumentative tendencies, increased anxiety and tension, insomnia, and decreased altruistic behavior. Traffic noise, indoor noise, etc., are some of the main causes of stress [2,3]. The human brain undergoes many physiological changes when responding to environmental stimuli [16]. Neural activity is promoted by glucose metabolism, and as neural activity increases, the consumption of glucose and oxygen in the local capillary layer increases, which causes changes in cerebral blood flow and cerebral oxygen metabolism. At this time, since the change in cerebral oxygen metabolism is small compared to the amount of oxygen supplied, the concentration of oxidized hemoglobin (HbO) in the blood increases, and the concentration of deoxygenated hemoglobin (HbR) decreases. Through this method, it is possible to observe how certain parts of the prefrontal cortex, which is the brain region that performs high-level processing and judgment planning, are activated when the brain is exposed to noise conditions. In addition, it has been shown that the prefrontal cortex’s function decreases under stress conditions, and blood flow tends to increase under negative emotions and stress conditions [17]. These investigations can provide basic data for identifying the physiological mechanisms involved in the response to noise exposure and developing better noise management and stress reduction technologies.

Previous studies [18,19,20,21,22,23,24,25,26,27,28,29,30,31] have suggested that noise can cause general psychological stress and cognitive decline, and policies have been proposed to reduce noise damage in specific environments. However, no specific railway environmental noise stress models have been developed according to altitude above, and distance from, railways. For example, it has been reported that traffic noise in a large city can cause an increased heart rate and increased secretion of stress hormones, although these results were obtained from a subjective survey. Accordingly, research that can specifically visualize and quantify physiological responses is necessary. This would enable the impact of noise on the human body to be minimized from the design stage and would also provide important basic data for establishing railway design strategies for urban development and improving the quality of life of citizens.

This study monitored brain activity changes in real time using an fNIRS technique in an objective manner. Functional near-infrared spectroscopy is a method that can noninvasively measure changes in blood oxygenation in the cerebral cortex and is advantageous in understanding the effects of environmental noise on specific areas of the brain. In this study, we hypothesized that the level of stress felt by people would differ depending on their specific distance from and altitude with respect to environmental noise, considered environmental conditions categorized according to this hypothesis, confirmed the level of stress through brain activity analysis, and identified which brain regions were affected. This is the first attempt to determine the areas that are stressed by environmental noise.

Even outside the railway field, there are studies using EEG in relation to noise effects, and EEG is a method to measure electrophysiological brain activation, while fNIRS is a method to measure hemodynamic responses. fNIRS is less expensive than fMRI, and the equipment that it requires does not produce noise and is more portable than that used for EEG, which makes it suitable for quantifying environmental variables [19,20,22,24,26,27,28,31,32,33,34,35,36].

This study provides fundamental scientific insights for optimizing comfort in railway vehicles, with the ultimate goal of enhancing the overall satisfaction of both railway passengers and nearby residents. The findings also offer practical guidance for refining the quality of railway services, contributing to a more pleasant travel experience. The data will contribute to future improvements in the quality of public transportation services and user-centered space design. The results will also be used to strengthen the overall competitiveness of the public transportation system, enabling the provision of better services.

## 2. Materials and Methods

### 2.1. Experimental Conditions and Settings

**Figure 1:**
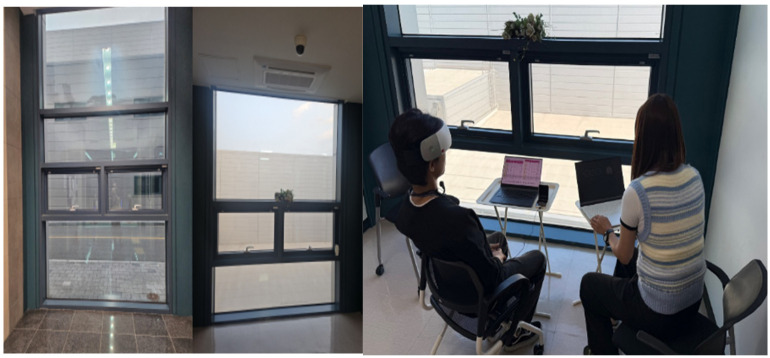
The railway environmental noise test setup (altitude, 1st and 4th floor; distance, 40 and 100 m).

The experiments were conducted on the 1st and 4th floors of a building in which the environmental variables could be controlled. The building was situated alongside a high-speed railway line. To minimize the impact of railway environmental noise, the space in which the measurements were made and the waiting area were deliberately separated. The laboratory conditions were carefully controlled, with a consistent temperature of 26 °C and relative humidity of 60% (as shown in Figure 1). Within the measurement area, a 16-inch monitor was positioned at a comfortable eye level for the participants (Figure 1).

### 2.2. Experimental Design

The experiment was conducted on 1 October 2024, with 10 participants. This number of participants was based on previous research, to ensure manageable waiting and testing times. The experiment involved two altitudes (1st and 4th floor of a building) and distances from the railway (40 and 100 m). The reason why the distance was set to 40 m and 100 m is because the shortest established distance between a building and a railway site is 40 m, and the maximum distance is 100 m. Also, the height was set to the 1st and 4th floors of a building to reflect the fact that the highest noise barrier in this study was approximately 12 m.

**Figure 2:**
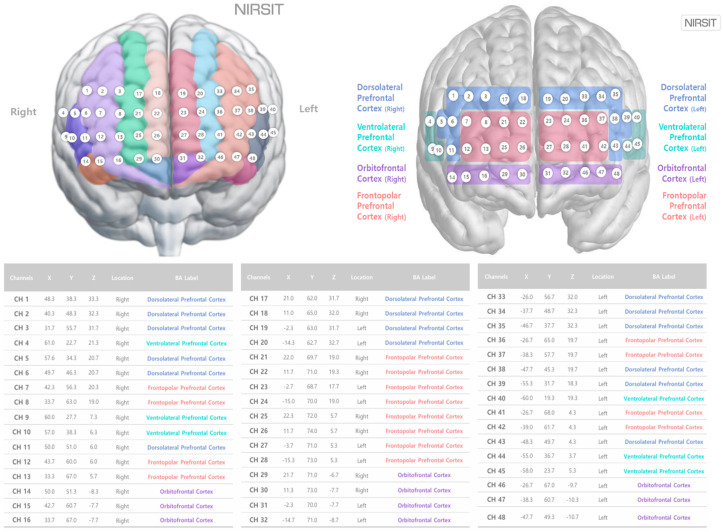
48 Channels and Brodmann mapping of NIRSIT channels.

The experimental procedure was as follows. The researcher introduced the experimental procedure to the subject, and the subject signed a consent form. The subject waited in the waiting room and later participated in the experiment at the designated testing location. The participant sat on a chair in front of the monitor in the study area, and the researcher prepared the environment according to the required experimental conditions. To measure the activity in the prefrontal cortex of the brain, a NIRSIT Lite system (OBELAB, Seoul, Republic of Korea) operating with 48 channels was securely fixed at the center of the participant’s forehead, 1 cm above their eyebrows. Each channel measured the optical signal from a specific region of the prefrontal cortex. The participant’s hair was pulled back as much as possible and fixed so that no light could enter the device prior to the measurements. When using this method, the 48 channels correspond to regions in the left and right frontal lobes as shown in Figure 2, and the Brodmann area is divided into left and right portions, including dorsolateral prefrontal cortex, ventrolateral prefrontal cortex, orbitofrontal cortex, and frontopolar prefrontal cortex (OBELAB).

**Figure 3:**
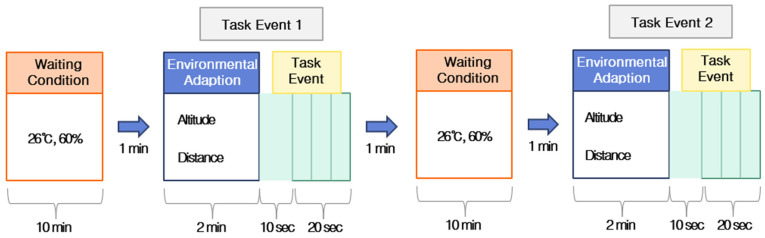
Design of the experimental process.

During the experiment, calibration was performed for each subject, and each participant was advised to maintain a comfortable body and head posture and to refrain from head movement as much as possible. The experimental data were recorded using the NIRSIT SCAN ver. 1.3 software (OBELAB). The experimental design is shown in Figure 3. Prior to the measurements, the participants were advised to rest comfortably in the waiting room for 10 min. The NIRSIT Lite equipment was then fixed at the testing site, and the participants were asked to close their eyes for 1 min to stabilize their brain activity. A different 2 min stimulus was then provided depending on the altitude and distance from the railway, and a task was performed for 30 s. The same test was repeated twice. The task was designed to require approximately 30 s per question based on a preliminary assessment and was designed to be moderately challenging but not overly difficult. The task showed guide maps frequently seen in railway stations and subways and presented 3 questions related to the information on the guide maps. The questions were presented intermittently only during a 30-s interval in the task event section, as shown in Figure 3. For the task, if the statement in the question was true, the subjects were asked to indicate OK with their fingers, while if it was false, they were asked to draw a V. The questions were asked randomly and regarded whether the waiting room was on the basement level, whether the subway map had information about tunnel evacuation routes, and when the first train would leave. This procedure was repeated, and the experiment was conducted twice at each altitude and distance, resulting in a total duration of approximately 10 min per experiment (Figure 3). To capture the noise levels in the study area, we recorded the sound five times as a train passed using a Samsung Galaxy Z Flip 5 smartphone, which featured Dolby Atmos 3D surround sound technology. This device provided a precise and immersive audio recording of the on-site environment.

### 2.3. Features of the fNIRS Test Device

Using fNIRS when analyzing the effects of the environmental variable of railway noise, specific areas of the cerebral cortex that influence passenger comfort can be identified. This approach considers both the environmental conditions and value judgments that affect comfort, as well as the brain activity patterns associated with relaxation. The ultimate aim of the study was to ensure the optimal design of new railway developments, enhancing the comfort of the residents living in and using the surrounding areas. Magnetic resonance imaging (MRI) studies provide high-resolution images but are expensive and time-consuming, while electroencephalography (EEG) is a neurophysiological measurement method that is relatively inexpensive and portable. Some previous studies have applied this method to measure brain surface activity. Functional near-infrared spectroscopy offers the added benefit of monitoring hemodynamic changes in real time by irradiating the cerebral cortex with two wavelengths of near-infrared light (780 and 850 nm) [32,37,38,39,40,41,42,43,44,45,46]. Infrared spectral light has the unique property of passing through tissue and being preferentially absorbed by hemoglobin (Hb) in the cerebral cortex [47,48]. The absorbance spectrum of Hb changes, depending on the extent to which it is bound to oxygen. This allows fNIRS to monitor changes continuously in oxyhemoglobin (HbO) and deoxyhemoglobin (HbR) concentrations within cortical regions, providing valuable insights into brain activity and oxygenation [48,49]. Based on the phenomenon of neurovascular coupling, the fNIRS signal is considered to be a surrogate measure of basal neural activity [48,50]. Local neural activity induces increases in blood flow and volume that are several times higher than the metabolic demand. Therefore, cerebral hemodynamic responses are typically accompanied by a large increase in HbO and a small decrease in HbR [48,51]. Because there is a net change in Hb state, HbO is used as a marker of brain activity [48,52]. Near-infrared light cannot reach subcortical brain regions, but its use is noninvasive and enables brain activity to be mapped by measuring changes in Hb concentration [48,53].

### 2.4. Participants

Ten male college students participated in the study (mean age 33.2 ± 5.6). According to the National Statistical Portal (KOSIS, 2023), ‘Railway Engineers and Related Workers’ include 0.9% of females, indicating that the railway industry mainly involves men; the experimental group was composed of men also to minimize physiological differences between the subjects. A similar study [19] was conducted on 12 men to evaluate car interior noise using brain waves.

All participants were physically healthy, with no history of disease. Individuals presenting with diseases such as COVID-19 on the day of the preliminary investigation and examination were excluded from the experiment. In addition, those with hearing discomfort, a history of neurological disease and sensory abnormality, and those with difficulties in undertaking physical activity or communication were excluded. Previous studies [19,20,22,24,26,27,28,31,32] using an EEG involved at least 10 and at most 25 participants, and therefore this study had an appropriate sample size. The number of subjects required to achieve the research objective was calculated using the G*power program 3.1.9.7. Based on the calculation results, for a significance level of 0.05, a power of 0.95, and an effect size 0.3, a minimum of 8 research participants was required [54,55]. The experimenter wore short sleeves and shorts, as the experiment was conducted in summer. Prior to the work commencing, the study proposal was reviewed by the joint institutional bioethics committee in accordance with the guidelines of the Declaration of Helsinki (P01-202409-01-060).

### 2.5. Pre-Procedure

Data measurement and analysis were performed using NIRSIT Quest ver. 1.1.2 software (OBELAB). Quantitative trends in the degree of activation of the frontal lobe were obtained based on the exposure to railway environmental noise on each floor and at each distance, and the degree of activation was derived with images based on t-values. Signal preprocessing was performed on the results using NIRSIT SCAN. Preprocessing procedures were performed in the following order: handling invalid values, channel rejection, data conversion, motion artifact removal using temporal derivative distribution repair (TDDR), data conversion, digital filtering, channel rejection, and rejection padding. For handling invalid values, a nearest-neighbor interpolation was applied if there were no more than five invalid cases in a row. The channels were rejected if there were more than five cases. Channel rejection was performed on raw intensity data, as follows. Channels with a median intensity lower than 30 arbitrary units (A.U.) and with a coefficient of variation greater than 15% were rejected. Channels with the same consecutive values that accounted for more than 5% of the entire time series (indicating saturation) were also rejected. Data conversion was performed on the raw intensity data by converting light intensity into optical density. Motion artifact removal using TDDR was performed on the Hb concentration data by conducting a temporal derivative distribution repair. Data conversion was performed by converting optical density to Hb concentration data based on International Organization for Standardization (ISO) guidelines. Concentration changes were calculated without accounting for different path lengths. The molar extinction coefficients calculated by Moaveni et al. (1970) [56] were used in this study. The Hb concentration unit was mm·mM. Digital filtering was performed on the Hb concentration data to eliminate irrelevant frequency components. Specifically, a discrete cosine transform (DCT) bandpass filter (low cutoff frequency of 0.005 Hz and high cutoff frequency of 0.1 Hz) was used to remove potential physiological noise, measurement noise, and drift. Channel rejection was performed for the Hb concentration data by rejecting channels whose HbO and HbR exhibited an extreme negative correlation (same graph form as mirroring) of −0.9 or higher. Finally, rejection padding was performed to handle missing or noisy data.

Specifically, the values from the backup channels were replaced with the mean values of the corresponding Brodmann area group. If no backup channels were available, the mean value of all remaining channels was used instead. In this study, 48 channels at a distance of 3 cm from the detector were targeted, and there was a total of 68 channels, including a backup channel below each channel. A rigorous data quality standard was maintained, with an average channel rejection ratio of less than 5%.

### 2.6. Procedure

Three categories of results can be obtained using fNIRS: block averaging, general linear model (GLM), and connectivity. This study employed a GLM approach, which provided a comprehensive framework for estimating the effects of multiple factors while controlling for other variables and accounting for individual differences. The GLM analysis was conducted at both the individual subject and the group levels, allowing the complex interplay of various influences on the data to be examined. At the subject level, signal normalization was not applied to maintain the original effects at the group level. Instead, the autoregressive iteratively reweighted least squares (AR-IRLS) procedure was used to remove autocorrelation, taking into account the extreme values present in the data. For the derivative, a combination of hemodynamic response function (HRF), temporal derivative, and dispersion derivative was chosen. In the nuisance regressor, the angles x, y, and z were included, and a shot separation channel (≤15 mm) was determined by channel mean correction, to minimize the confounding effects.

For the group-level analysis, given the 2 × 2 factorial design of the conditions (altitude: 1st vs. 4th floor; distance: 40 vs. 100 m), a paired-sample *t*-test was employed to examine the group-level statistics and generate activation maps. In this study, the paired-sample *t*-test was performed as a statistical method to test whether there was a significant difference between the means at different points in the same sample group. The null hypothesis was that there would be no difference, and the alternative hypothesis was that there would be a difference. In this study, the paired-sample *t*-test was used because the number of participants was small, and using a proven statistical method can increase the reliability of the interpretation. In addition, in this study, the FDR (false discovery rate) method was used to check the average proportion of falsely rejected hypotheses among the rejected null hypotheses.

This approach allowed us to identify significant differences in brain activity between the different altitude and distance conditions.

## 3. Results

### 3.1. The Impact of Altitude on Railway Environmental Noise

The paired-sample *t*-test analysis for altitude was conducted using NIRSIT Quest ver. 1.1.2 software, employing a one-way technique. The results revealed significant differences in HbO concentrations according to the floor level in response to railway environmental noise. Specifically, the HbO concentration by channel was found to be −0.009 ± 0.045 mm·mM on the 4th floor and −0.009 ± 0.040 mm·mM on the 1st floor. Similarly, the HbO concentration by Brodmann area was −0.010 ± 0.030 mm·mM on the 4th floor and −0.011 ± 0.026 mm·mM on the 1st floor. We observed an overall negative value, indicating a relative decline in the HbO concentrations compared to the typical levels. Small activation does not mean that there is no brain activity, but that the activation is smaller under the given condition with respect to the baseline levels. In the Brodmann area, the HbO concentration was −0.010 ± 0.030 mm·mM in layer 4 and −0.011 ± 0.026 mm·mM in layer 1, confirming that layer 4 was relatively more activated. In the case of the frontal lobe, it was seen that it was relatively stressed under the layer 4 condition, as its function decreases in times of stress, and blood flow tends to increase in times of negative emotion and stress [17].

Furthermore, we found that the degree of activation varied according to the cerebral hemodynamic response. The results of the paired-sample *t*-test analysis performed with two samples and one independent variable are shown in Table 1, Table 2, Table A1 and Table A2.

**Figure 4:**
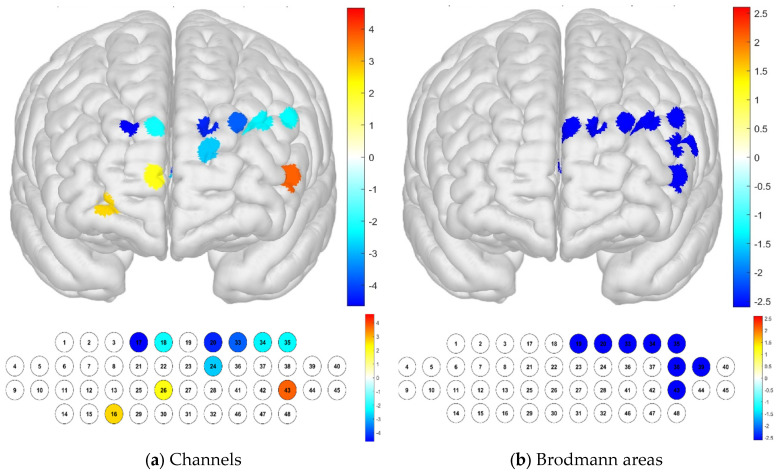
Brain activation analysis in different channels (**a**) and Brodmann areas (**b**) based on *t*-test results at different altitudes (paired-sample Student’s *t*-test results for HbO concentration, *p*-value from Student’s *t*-test (uncorrected *p* < 0.05)).

Notably, significant differences were found in channels 16, 17, 18, 20, 24, 26, 33, 34, 35, and 43 when analyzing the layers. Specifically, channel 43 exhibited high levels of brain activation, whereas channels 17, 18, 20, 24, 33, 34, and 35 displayed lower sample averages, indicating lower levels of brain activation. In the Brodmann area, significant differences were confirmed in channels 19, 20, 33, 34, 35, 38, 39, and 43, and all areas had low sample averages, indicating low levels of brain activation. In particular, activation was found to be significant in the left DLPFC (*p* < 0.05) (Table 1; Figure 4). The DLPFC area in the frontal lobe plays a role in task-oriented and reasoning abilities.

### 3.2. The Impact of Distance on Railway Environmental Noise

The paired-sample *t*-test analysis for distance was conducted using NIRSIT Quest ver. 1.1.2 software, employing a one-way technique. Considering the impact of distance on the response to railway environmental noise, we found that the HbO concentration by channel was −0.008 ± 0.043 mm·mM at 40 m and −0.009 ± 0.046 mm·mM at 100 m. Similarly, the HbO concentration by Brodmann area was −0.010 ± 0.027 mm·mM at 40 m and −0.010 ± 0.030 mm·mM at 100 m. The overall negative value indicated a decline in the HbO concentrations compared to the typical levels. Small activation does not mean no brain activity, but rather that activation is small under the given conditions. The channel-wise HbO concentrations were −0.008 ± 0.043 mm·mM at 40 m and −0.009 ± 0.046 mm·mM at 100 m, confirming that there was more activation at 40 m. In the case of the frontal lobe, it can be seen that it was relatively stressed under the 40 m condition, as its function decreases in times of stress and blood flow tends to increase in times of negative emotion and stress [17]. Especially, we observed a variation in the degree of brain activation according to the cerebral hemodynamic response. The results of the paired-sample *t*-test analysis performed with two samples and one independent variable are shown in Table 3, Table A3 and Table A4.

**Figure 5:**
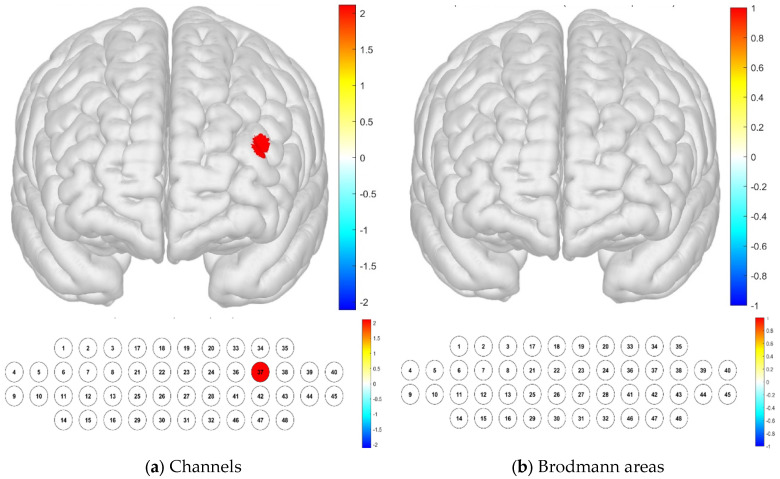
Brain activation analysis in different channels (**a**) and Brodmann areas (**b**) based on *t*-test results at different distances (paired-sample Student’s *t*-test results for HBO concentration, *p*-value from Student’s *t*-test (uncorrected *p* < 0.05)).

Regarding the differences in brain activation according to the distance, it was confirmed that there was a significant difference in channel 37, which had a particularly high level of activation. In the Brodmann area, it was confirmed that there was no significant difference in the channels in the entire area (*p* < 0.05) (Figure 5).

### 3.3. t-Test by Detailed Condition

To investigate the degree of brain activation for each specific condition, this study employed one-sample *t*-tests to analyze the data individually for each of the four conditions: two conditions per floor (1st and 4th floor) and two conditions per distance (40 and 100 m).

**Figure 6:**
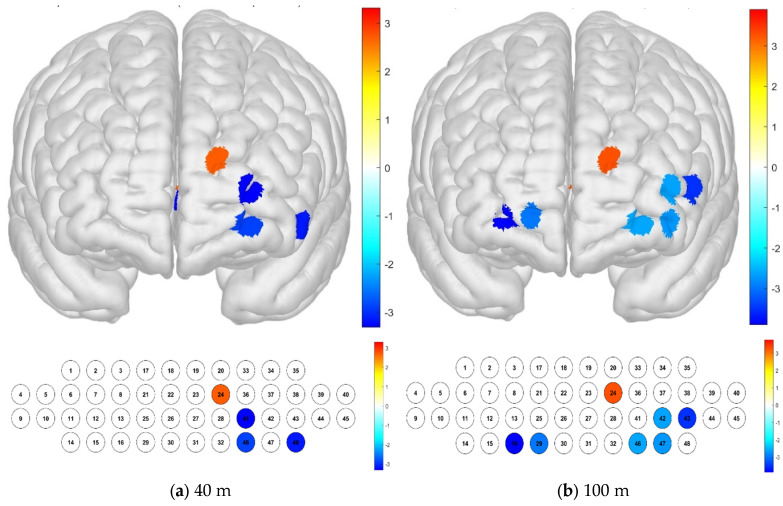
Brain activation with respect to the distance from the railway on the 1st floor: *t*-test results by channel (paired-sample Student’s *t*-test results for HbO concentration, *p*-value from Student’s *t*-test (uncorrected *p* < 0.05)). (**a**) Distance of 40 m, (**b**) distance of 100 m.

At a distance of 40 m from the railway on the 1st floor, the sample average was lowest in channels 41, 46, and 48, confirming low levels of activation, while in channel 24, there was a high level of activation. At a distance of 100 m on the 1st floor, the sample average was lowest in channels 16, 29, 42, 43, 46, and 47, confirming a low level of activation, while in channel 24, there was a high level of activation. Regardless of the distance, it was confirmed that the level of activation was high in channel 24 (Figure 6).

**Figure 7:**
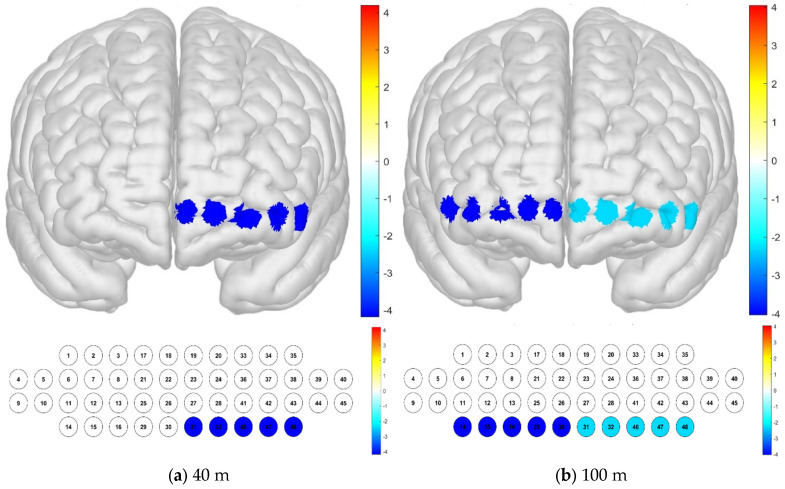
Brain activation with respect to the distance from the railway on the 1st floor: *t*-test results by Brodmann area (paired-sample Student’s *t*-test results for HbO concentration, *p*-value from Student’s *t*-test (uncorrected *p* < 0.05)). (**a**) Distance of 40 m, (**b**) distance of 100 m.

An examination of the Brodmann area confirmed that the left OFC area had a low level of activation at a distance of 40 m from the railway on the 1st floor, and the OFC area in the entire left and right regions had a low level of activation at a distance of 100 m from the railway on the 1st floor. The OFC is a region related to emotional regulation and reward processing (Figure 7).

**Figure 8:**
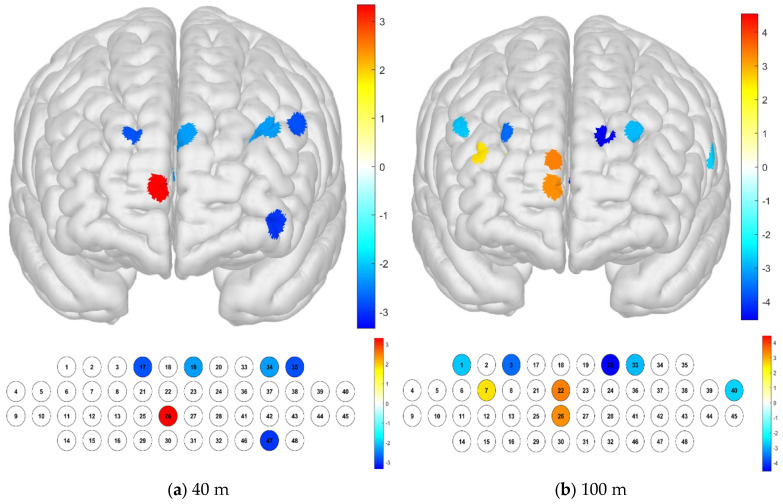
Brain activation with respect to the distance from the railway on the 4th floor: *t*-test results by channel (paired-sample Student’s *t*-test results for HbO concentration, *p*-value from Student’s *t*-test (uncorrected *p* < 0.05)). (**a**) Distance of 40 m, (**b**) distance of 100 m.

At a distance of 40 m from the railway on the 4th floor, channels 17, 19, 34, 35, and 47 had much lower sample averages than the other channels, confirming low levels of activation, while channel 26 had a high level of activation. At a distance of 100 m from the railway on the 4th floor, channels 1, 3, 20, 33, and 40 had much lower sample averages than the other channels, confirming low levels of activation, while channels 22 and 26 had high levels of activation. Regardless of the distance, it was confirmed that the level of activation was high in channel 26 (Figure 8).

**Figure 9:**
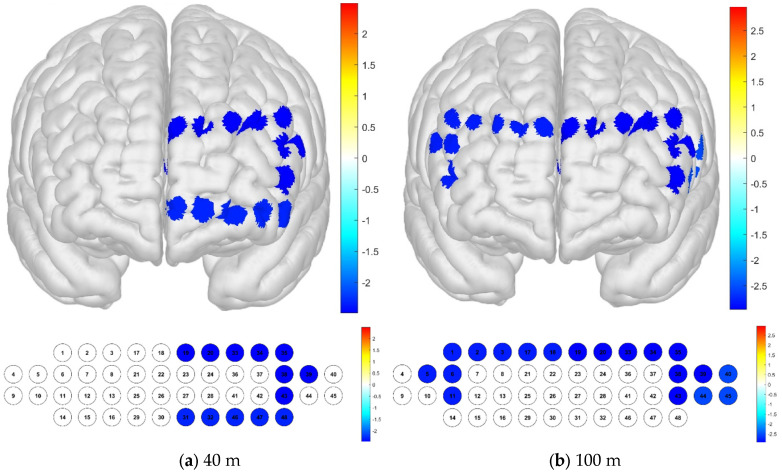
Brain activation with respect to the distance from the railway on the 4th floor: *t*-test results by Brodmann area (paired-sample Student’s *t*-test results for HBO concentration, *p*-value from Student’s *t*-test (uncorrected *p* < 0.05)). (**a**) Distance of 40 m, (**b**) distance of 100 m.

An examination of the Brodmann area confirmed that the left OFC and left DLPFC areas had low levels of activation on the 4th floor at a distance of 40 m from the railway. The left and right DLPFC in their entirety had low activation levels on the 4th floor at a distance of 100 m from the railway. The OFC is a region particularly related to emotional regulation and reward processing, while the DLPFC is a region related to work and reasoning ability. It could be concluded that railway environmental noise has an emotional impact at relatively close distances to the railway (Figure 9).

## 4. Discussion

Railway construction and operation result in numerous environmental concerns, with noise generation from operational routes being a persistent issue. This issue generates widespread complaints, which ultimately result in significant social and economic costs. Noise is one of the main complaints resulting from railway construction and operation and, although there is a proposal for all new railway developments to be located at a distance of 100 m from residential buildings, no specific distance standard has actually been provided. Additionally, the installation of noise barriers is a common measure used to reduce railway environmental noise, although a scientifically established standard height has not been set in Korea. To assist with the setting of these standards, our study aimed to determine the optimal conditions for railway environmental noise reduction by analyzing changes in blood flow in the cerebral cortex in response to noise perceived at varying distances from and altitudes above a railway by individuals living in the vicinity of the railway. While previous studies mainly focused on the noise produced by railway vehicles themselves and the possible reduction measures, most have been based on subjective evaluations based on questionnaires. This study quantitatively analyzed the results of physiological responses based on an fNIRS analysis. fNIRS uses brain signal characteristics based on oxygenated hemoglobin concentration determined in different research conditions and enables a classification of the physiological responses by behavior such as task and rest; it is meaningful in that it can classify different responses in each individual.

fNIRS measures the activity in the prefrontal cortex and can quantify stress [52,53,57,58,59,60]. fNIRS is a cognitive neuroscience technology that reveals how thought processes, perception, memory, and emotions expressed in the brain proceed, through brain activity data. It can observe how certain parts of the prefrontal cortex are activated by noise and aims to find significant differences in the average activation values by brain region.

There is a tendency for blood flow to decrease in the frontal lobe in response to positive emotions, while blood flow increases in response to negative emotions and stress. The HbO concentrations under the different railway environmental noise exposures depending on the distance from and the altitude above the railway were generally negative. It was confirmed that more relative discomfort was felt at 40 m from the railway than at 100 m. Additionally, it was confirmed that similar feelings of discomfort were felt on both the 1st and the 4th floors.

This pioneering study marks the first application of fNIRS to a railway environmental noise environment. In particular, traffic noise is one of the major causes of stress, and it has been suggested that exposure to high-intensity noise is associated with psychiatric symptoms [2,3]. Notably, our research makes a significant contribution to this field by identifying the specific channels and Brodmann areas affected by railway noise, with a particular focus on the prefrontal lobe of the brain, which was divided into left and right areas for a more detailed analysis. In particular, when looking at the results of the one-sample *t*-test, it was confirmed that activation was high in channel 24 regardless of the noise levels at different distances on the first floor. In the same locations, the OFC, which is related to emotional regulation, had a low level of activation. Additionally, it was confirmed that activation was high in channel 26 regardless of the noise levels at different distances on the 4th floor. For the Brodmann area, the left OFC and left DLPFC areas had low levels of activation at 40 m from the railway, and the DLPFC area had low levels of activation in the 100 m condition. The prefrontal lobe is a large region located at the front of the brain that plays a role in processing cognitive functions and emotional responses. The functioning of the prefrontal lobe decreases under stress. It is an area of the brain where blood flow tends to decrease under positive emotions and increase under negative emotions and stress [52,53,57,58]. The prefrontal cortex can be divided into four regions: the dorsolateral prefrontal cortex (DLPFC), the ventrolateral prefrontal cortex (VLPFC), the frontopolar prefrontal cortex (FPC), and the orbitofrontal cortex (OFC). The DLPFC is responsible for working memory and reasoning ability, and the VLPFC is responsible for working memory, language ability, and attention. The FPC is related to metacognition and decision-making. The OFC is involved in emotional regulation and reward processing [59,60,61].

Despite being a highly reliable indicator, few studies have explored how brain function responds to railway environmental noise at different distances from, and altitudes above, railway lines. It could be confirmed, therefore, that railway environmental noise has an emotional impact at relatively close distances to a railway. Our results are meaningful in that they quantitatively confirmed the relationship between stress and physiological responses due to traffic noise. The comfort levels of both local residents and passengers in response to railway environmental noise could therefore be improved by considering the brain areas with affected activation and the functions of each channel.

In addition, this study differs from other studies in that it conducted a paired-sample *t*-test based on altitude and distance from the railway and a one-sample *t*-test for each condition to derive the results. Based on the results of high or low activity in specific channels and Brodmann areas, it derived noise conditions associated with feelings of relative stress and comfort. Based on these results, it can be optimally determined how far away to install noise barriers and how high to set them, depending on railway noise and the location of major residential areas.

## 5. Conclusions

The paired-sample *t*-test analysis revealed distinct physiological response patterns in the different conditions examined. Notably, brain activation was higher in channel 43 when the participating individuals perceived differences in environmental noise by floor. Conversely, brain activation was lower in channels 17, 18, 20, 24, 33, 34, and 35. Furthermore, a significant difference was identified in the left DLPFC, a region known to be involved in working memory and reasoning ability. This finding suggests that environmental noise affects cognitive processes related to memory and reasoning. It was also confirmed that brain activation was high in channel 37, which is the channel responsible for sensing differences in environmental noise by distance, although there was no significant difference in the Brodmann area.

Through a quantitative analysis of the effects of railway environmental noise, we confirmed that changes in the activity of the prefrontal cortex of the brain according to distance from, and altitude above, a railway can be determined through a statistical analysis. We confirmed that the scientific analysis method that we used, based on experimental measurements, can supplement subjective and qualitative results based on questionnaires.

In particular, in studies based on fNIRS, it is necessary to perform a static calibration for measuring blood flow in the cerebral cortex. It is essential to fix the measuring apparatus securely to an individual, and it is also important to minimize head movement during the measurement, to ensure that the signal strength is not affected. To ensure good signal data, subjects should keep their head as still as possible when being questioned and during testing in noise conditions. Even with calibration, head movement may occur during tasks or rest, which may require additional processing of unnecessary signals and systematic supplementation to limit movement. In the future, we plan to improve potential preprocessing or motion compensation methods such as wavelet filtering, PCA, and spline interpolation. Through this, we aim to improve the performance of signal recognition. In addition, it will be necessary to additionally simulate in advance the environment for the participating subjects. In this study, some head movements limited signal processing.

In addition, although most of the participants in this study were men in their 30s, there are various characteristics that can be derived from the interpretation of the results in relation to women, age, health status, long-term noise exposure, etc.; so continuous research is needed. Therefore, we plan to conduct additional experiments considering gender, age, etc. As this is an initial study, securing basic data was the priority, and we plan to secure a lot of data considering various variables.

To analyze the stress from railway environmental noise exposure in local residents, we considered their distance from (40 and 100 m) and altitude above (1st and 4th floor) the railway. In the future, we will construct a basic database by examining a more diverse range of conditions. These results may contribute to urban planning and noise mitigation strategies.

Additionally, it is expected that it will be possible to expand and enhance the analysis of human stress based on various environmental variables, such as odor, temperature and humidity, and light annoyance, and it will then be possible to suggest ways for evaluating stress and comfort under various conditions.

## Figures and Tables

**Table 1:** The *t*-test analysis results by channel and floor.

Index	Student’s *t*	df	FDR-Adjusted *p*-Value
Channel 16	2.691	13	0.019 *
Channel 17	−4.651	19	0.000 *
Channel 18	−2.210	15	0.043 *
Channel 20	−4.315	19	0.000 *
Channel 24	−2.783	15	0.014 *
Channel 26	2.152	17	0.046 *
Channel 33	−3.768	17	0.002 *
Channel 34	−2.385	17	0.029 *
Channel 35	−2.319	15	0.035 *
Channel 43	3.782	19	0.001 *

(* *p* < 0.05).

**Table 2:** Results of the *t*-test analysis of Brodmann area by floor.

Index	Student’s *t*	df	FDR-Adjusted *p*-Value
Left_DLPFC	−2.603	19	0.017 *

(* *p* < 0.05).

**Table 3:** The *t*-test analysis results by distance and channel.

Index	Student’s *t*	df	FDR-Adjusted *p*-Value
Channel 37	2.113	19	0.048 *

(* *p* < 0.05).

## Data Availability

The original contributions presented in this study are included in the article. Further inquiries can be directed to the corresponding authors.

## Appendix A

**Table A1:** The *t*-test analysis results by channel and floor.

**Index**	**Student’s *t***	**df**	**FDR-Adjusted *p*-Value**
Channel 01	−1.341	13	0.203
Channel 02	0.377	15	0.712
Channel 03	−1.845	19	0.081
Channel 04	−0.608	13	0.554
Channel 05	−0.760	13	0.461
Channel 06	1.116	13	0.284
Channel 07	2.039	13	0.062
Channel 08	0.610	17	0.550
Channel 09	−1.224	13	0.243
Channel 10	0.244	17	0.810
Channel 11	1.274	19	0.218
Channel 12	−0.283	17	0.781
Channel 13	1.104	11	0.293
Channel 14	−0.258	9	0.802
Channel 15	−1.343	17	0.197
Channel 16	2.691	13	0.019 *
Channel 17	−4.651	19	0.000 *
Channel 18	−2.210	15	0.043 *
Channel 19	−1.902	19	0.072
Channel 20	−4.315	19	0.000 *
Channel 21	−0.061	19	0.952
Channel 22	0.606	17	0.553
Channel 23	−0.973	17	0.344
Channel 24	−2.783	15	0.014 *
Channel 25	1.084	11	0.301
Channel 26	2.152	17	0.046 *
Channel 27	−0.392	17	0.700
Channel 28	2.053	15	0.058
Channel 29	1.714	11	0.115
Channel 30	1.460	13	0.168
Channel 31	1.965	15	0.068
Channel 32	−0.252	15	0.804
Channel 33	−3.768	17	0.002 *
Channel 34	−2.385	17	0.029 *
Channel 35	−2.319	15	0.035 *
Channel 36	1.381	19	0.183
Channel 37	0.183	19	0.857
Channel 38	1.676	17	0.112
Channel 39	−2.039	17	0.057
Channel 40	−0.897	13	0.386
Channel 41	−0.186	17	0.855
Channel 42	1.163	19	0.259
Channel 43	3.782	19	0.001 *
Channel 44	−0.230	19	0.820
Channel 45	0.300	19	0.768
Channel 46	−0.255	15	0.802
Channel 47	−0.575	17	0.573
Channel 48	1.986	19	0.062

(* *p* < 0.05).

**Table A2:** Results of the *t*-test analysis of Brodmann area by floor.

**Index**	**Student’s *t***	**df**	**FDR-Adjusted *p*-Value**
Left_DLPFC	−2.603	19	0.017 *
Right_DLPFC	−1.053	19	0.305
Left_FPC	0.204	19	0.840
Right_FPC	1.664	19	0.112
Left_OFC	1.622	19	0.121
Right_OFC	1.562	19	0.135
Left_VLPFC	−0.595	19	0.559
Right_VLPFC	−0.009	17	0.993

(* *p* < 0.05).

**Table A3:** The *t*-test analysis results by distance and channel.

**Index**	**Student’s *t***	**df**	**FDR-Adjusted *p*-Value**
Channel 01	0.395	16	0.698
Channel 02	0.253	17	0.803
Channel 03	0.487	19	0.632
Channel 04	−0.011	15	0.991
Channel 05	−0.539	16	0.598
Channel 06	−0.986	16	0.339
Channel 07	−1.656	15	0.118
Channel 08	−0.037	18	0.971
Channel 09	0.759	15	0.460
Channel 10	0.400	18	0.694
Channel 11	−1.052	19	0.306
Channel 12	0.642	18	0.529
Channel 13	−0.259	13	0.800
Channel 14	−1.058	13	0.310
Channel 15	−0.167	18	0.869
Channel 16	−0.069	14	0.946
Channel 17	0.106	19	0.916
Channel 18	0.897	16	0.383
Channel 19	−0.782	19	0.444
Channel 20	1.360	19	0.190
Channel 21	−1.796	19	0.088
Channel 22	−0.619	17	0.544
Channel 23	−0.734	18	0.473
Channel 24	−1.052	16	0.308
Channel 25	1.356	13	0.198
Channel 26	−1.123	18	0.276
Channel 27	1.487	18	0.154
Channel 28	0.292	15	0.774
Channel 29	0.517	13	0.614
Channel 30	0.185	15	0.856
Channel 31	−1.124	16	0.278
Channel 32	0.487	15	0.633
Channel 33	0.943	18	0.358
Channel 34	−0.481	18	0.636
Channel 35	0.187	16	0.854
Channel 36	−0.407	19	0.689
Channel 37	2.113	19	0.048 *
Channel 38	0.310	18	0.760
Channel 39	0.600	18	0.556
Channel 40	0.374	16	0.713
Channel 41	−0.250	18	0.805
Channel 42	−0.168	19	0.869
Channel 43	0.591	19	0.562
Channel 44	0.667	19	0.513
Channel 45	2.015	19	0.058
Channel 46	1.311	16	0.208
Channel 47	0.032	18	0.975
Channel 48	−0.337	19	0.740

(* *p* < 0.05).

**Table A4:** The results of a *t*-test analysis of the Brodmann area by distance.

**Index**	**Student’s *t***	**df**	**FDR-Adjusted *p*-Value**
Left_DLPFC	0.931	19	0.364
Right_DLPFC	0.285	19	0.779
Left_FPC	0.029	19	0.977
Right_FPC	−0.990	19	0.334
Left_OFC	−0.240	19	0.813
Right_OFC	−0.870	19	0.395
Left_VLPFC	1.220	19	0.237
Right_VLPFC	0.353	18	0.728
